# Double trouble: compounding effects of COVID-19 pandemic and antimicrobial resistance on drug resistant TB epidemiology in India

**DOI:** 10.3389/fpubh.2023.1305655

**Published:** 2023-12-06

**Authors:** Aliabbas A. Husain, Rajpal Singh Kashyap

**Affiliations:** Advanced Research Center, Central India Institute of Medical Sciences, Nagpur, India

**Keywords:** TB, MDR-TB, India, antimicrobial resistance, COVID-19

## Introduction

Antimicrobial resistance (AMR) has been regarded as a global health threat by the WHO (1). The rise of general AMR to multiple drugs in the Indian population poses a formidable challenge to the TB control strategies, especially for drug-resistant tuberculosis (DR-TB). India has been regarded as having one of the highest burdens of drug resistant (DR) pathogens, including multi-drug-resistant TB (MDR-TB), which accounts for a quarter of the global burden (2). A 6-year observational study in south India on the prevalence of DR-TB indicated a high proportion (11%) of rifampicin and isoniazid mono-resistance in new cases (3). Increasing trends of DR-TB in new cases highlight potential public health concerns, as globally, 78% of rifampicin-resistant TB (RR-TB) cases were identified to be MDR-TB (3). In India, major mediating factors for the high incidence of DR-TB in new cases include transmission of AMR strains in endemic communities and acquired DR due to misuse and overuse of antimicrobials.

## The COVID-19 pandemic and its consequences on TB care in India

A compounding effect of COVID-19 on TB care has been extensively reported earlier in various countries (4–6). According to a recent WHO global TB report, India was the largest contributor to the global shortfall in TB case notification in 2020 compared to other high-burden countries (7). A significant impact of reduced TB case notification was directly observed on the incidence of TB and DR-TB in India, which showed a significant decline in 2020 (8) compared to previous years, which should be mainly attributable to restricted access to healthcare facilities amid lockdown measures in the country (5, 9). Available reports suggest MDR-TB patients co-infected with COVID-19 pose formidable challenges in treatment, as COVID-19 can favor bacterial replication in the lungs through interference in intestinal homeostasis (10, 11). The empirical use of antibiotics is considered to be an important risk factor for the development of resistance (11–13). As per a recent modeling analysis report, COVID-19 in India contributed to about 216 million excess doses of antibiotics in between epidemic peaks (14). Reports from a meta-analysis by Langford et al. (15) showed high antibiotic usage (~71.9%) in hospitalized COVID-19 patients, even though only 6–9% of them were confirmed to have bacterial infection. Studies also indicate high antibiotic prescribing patterns of almost 82% in Southeast-Asian countries (excluding China) during the pandemic period (16). As a result, the incidence of MDR infections in COVID-19 patients is estimated to be around 32–50% (17). Additionally, oral glucocorticoids such as methylprednisolone and intravenous monoclonal antibody tocilizumab therapy to control IL-6-induced cytokine release syndrome (CRS) were used rampantly in India during the second wave, despite a stringent WHO advisory on their administration (18). A case report by Khayat et al. indicated that prolonged corticosteroid therapy in COVID-19 leads to significant CD4 and CD8 T-cell depletion and may promote the development of active infection in latent close contacts of TB cases (19). This represents a significant public health concern, particularly for COVID-infected close contacts of MDR-TB cases, who might be at high risk for conversion to active infection due to depletion and dysfunction of T-cells similar to HIV.

## Increasing AMR in India and its impact on MDR-TB epidemiology

**Figure 1:**
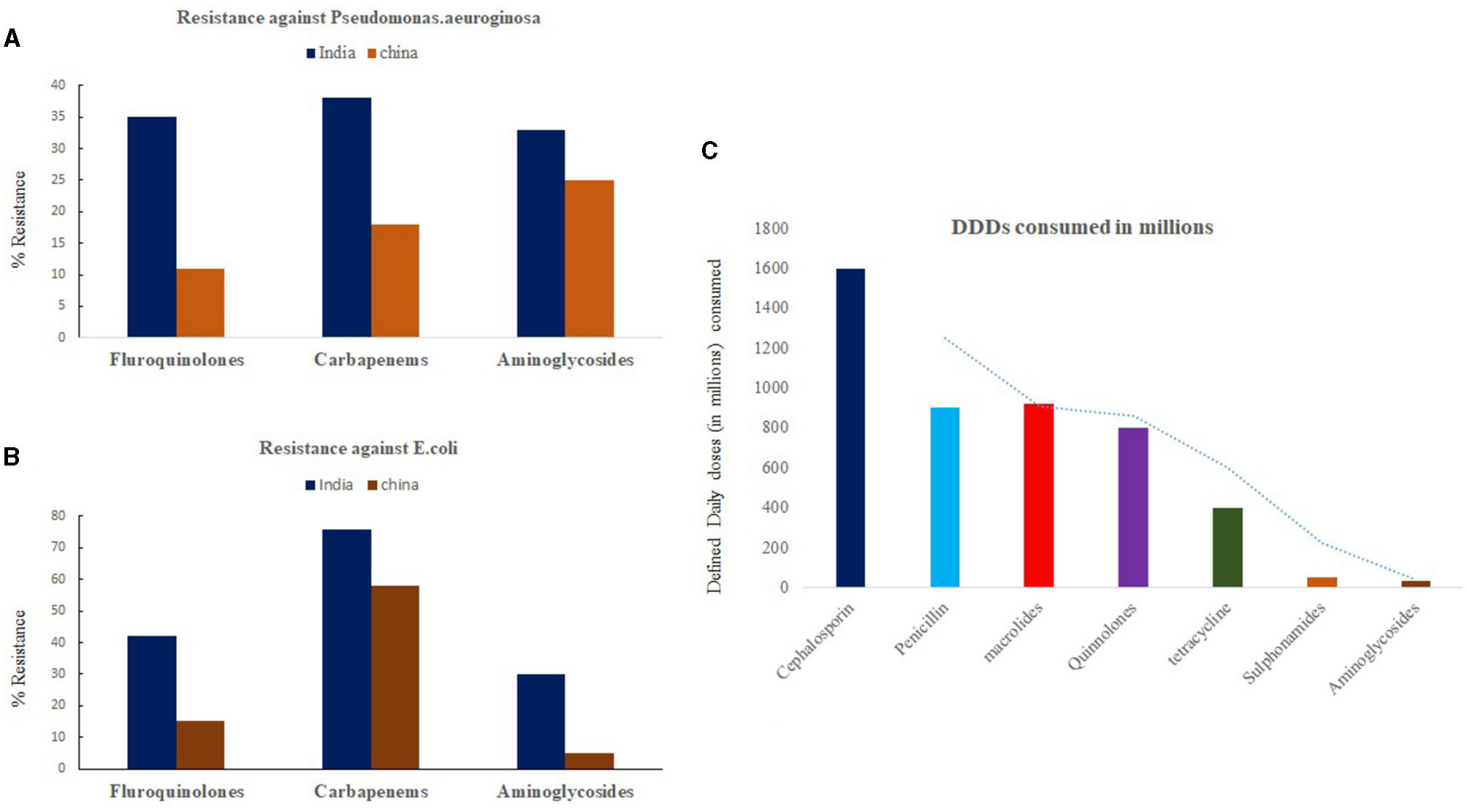
Trend of Antibiotic resistance (2019–20) among second line drugs against invasive isolates of **(A)** *Pseudomonas aeruginosa* and **(B)** *E. coli* in India and China. **(C)** Represents trend of antibiotic consumption pattern of common drugs in Indian population represented as defined daily dose (DDDs) per million populations. Source: One Health Trust. Resistance Map: Antibiotic resistance 2023. https://resistancemap.onehealthtrust.org/AntibioticResistance.php (accessed May 31, 2023).

India has been regarded as the global capital of AMR (2). According to the national anti-TB DR survey conducted by the Indian government in collaboration with the WHO and the United States Agency for International Development (USAID), it was found that around 23% of new cases showed resistance to any drug, with MDR-TB detected in 3% (20). In addition, ~36% of MDR-TB cases tested for resistance to second-line anti-tuberculosis medications showed resistance to fluoroquinolones (2). One of the major drivers of AMR in India is the irrational consumption of non-prescribed drugs taken as self-medication (21). India is regarded as the top consumer of antibiotics for humans among all other countries (22). Between 2005 and 2009, India witnessed a 40% rise in the sale of antibiotics, with sales of new-generation cephalosporin increasing by almost 60% (23). According to one survey, ~52% of the Indian population is estimated to be self-medicating (24). Despite regulations on the sale of schedule H1 and X drugs in India, second-line drugs like fluoroquinolones are readily available over the counter (OTC) in pharmacies due to their rampant empirical use against a wide variety of infections. Such OTC practices are even more prevalent in peripheral and rural zones of India, wherein mostly experienced and untrained pharmaceutical staff can prescribe the wrong combination and duration of drugs to the locals. As a result, it is estimated that the majority of people consuming OTC drugs generally under-dose themselves, exposing bacteria to non-lethal drug concentrations and thereby developing DR. Figures 1A, B shows general drug resistant pattern of carbapenem, fluoroquinolones and aminoglycosides on common invasive isolates of *E. coli, P. aeruginosa* in India and China. In addition to self-medication, high prescription and consumption patterns of second- and third-line antibiotics (Figure 1C) and mismanagement of TB presumptive cases by private physicians and pharmacists at the primary care level have been attributed to bolstering AMR cases in Indian communities (24). A study published by Arinaminpathy et al. (25) shows mismanagement and a lack of standard of care followed by private practitioners among presumptive TB cases as one of the major drivers for MDR-TB development. Similar studies by Kwan et al., based on referral rates of patients to healthcare centers, have shown a significant impact of urban pharmacies on TB control (26). Their study shows low referral rates of presumptive cases by urban pharmacies as a major compounding factor for low detection rates and disease control.

## Actions taken so far to combat AMR and DR-TB in India

To combat AMR, India introduced several measures, including the “Red Line” campaign in 2016 and the National Action Plan (NAP) on AMR in 2017 (27). Additionally, the Cosmetics and Drug Act launched in 2013 prohibited the sale of Schedule H1 drugs without prescription in India, which includes second- and third-generation antibiotics and anti-TB drugs. With due cognizance of AMR, the National Center for Disease Control under the Ministry of Health and Family Welfare, Government of India, developed a national plan on AMR containment with the objectives of improving awareness and surveillance of AMR in Indian health settings. In 2017, India launched its revised national strategic plan (NSP) with the ambitious goal of ending TB by 2025. Under its revised NSP (2017–25), the Government of India introduced several aggressive steps, such as decentralization of DR-TB services for better accessibility under the private sector, universal drug susceptibility testing (U-DST) for presumptive patients from MDR-TB hotspots, and scaling up diagnostic services of CBNAAT, True NAT, and line probe assays in low-resource settings for better surveillance and diagnosis (28).

## Discussion

Despite the aggressive efforts made under the revised NSP, DR-TB cases continue to bolster in Indian health settings. Improved commitments are needed to integrate antimicrobial stewardship programs under pandemic response to frontline providers for the management of TB patients co-infected with COVID-19. Integration of TB health services with AMR programs can be done to leverage the expertise of TB control planners for strengthening diagnostic capacity for AMR testing, surveillance, better quality assurance, record keeping, and logistics. Expanding the diagnostic scope of TB testing laboratories for AMR surveillance in high-endemic spots for TB can be useful for developing important public health measures for combating AMR. Joint efforts and funding commitments from major TB stakeholders are needed globally to develop improved diagnostics and surveillance tools to detect AMR in second- and third-line drugs in high-TB settings. Similarly, efforts are needed to develop diagnostic stewardship programs that include mandatory U-DST for second-line drugs in COVID health care settings, COVID-19 hotspots and TB-endemic settings for early diagnosis and treatment outcomes. Accelerated surveillance and vigilance at local pharmacies is needed to minimize the sale of OTC schedule H1 and X drugs. The development of an online, multilingual, free consultation system for primary care physicians with TB health care providers with expertise in clinical and management experience can improve AMR stewardship for DR-TB. The European respiratory society has a similar web-based free consultation system for South Africa (29), which can be extended to highly endemic South Asian countries for better DR-TB management.

The alarming rise of AMR in Indian communities in the aftermath of waves combined is a major risk driver for future MDR-TB epidemics in India. The country needs immediate attention on surveillance of DR in COVID hotspots to minimize future transmission of MDR in communities. Additionally, increased political commitments and advocacy are needed to bolster antimicrobial stewardship in COVID care hospitals and quarantine zones to minimize the irrational use of empirical regimens. Such advocacy is needed to prevent the risk of the development of AMR in Indian communities and improve treatment outcomes for cases of MDR-TB in India in the near future.

## Author contributions

RK: Writing – original draft, Writing – review & editing. AH: Conceptualization, Writing – original draft, Writing – review & editing.
